# Effect of a sulforaphane supplement on muscle soreness and damage induced by eccentric exercise in young adults: A pilot study

**DOI:** 10.14814/phy2.15130

**Published:** 2021-12-19

**Authors:** Shoichi Komine, Ikuru Miura, Nao Miyashita, Sechang Oh, Katsuyuki Tokinoya, Junichi Shoda, Hajime Ohmori

**Affiliations:** ^1^ Faculty of Human Care Teikyo Heisei University Toshima‐ku Japan; ^2^ Faculty of Medicine University of Tsukuba Tsukuba Japan; ^3^ Doctoral program in Sports Medicine Graduate School of Comprehensive Human Sciences University of Tsukuba Tsukuba Japan; ^4^ Master's program in Physical Education, Health and Sport Sciences Graduate School of Comprehensive Human Sciences University of Tsukuba Tsukuba Japan; ^5^ Department of Health Promotion Sciences Graduate School of Human Health Sciences Tokyo Metropolitan University Hachioji‐shi Japan; ^6^ Japan Society for the Promotion of Science Chiyoda‐ku Japan; ^7^ Faculty of Health and Sport Sciences University of Tsukuba Tsukuba Japan

**Keywords:** antioxidant supplement, DOMS, MDA, Nrf2, oxidative stress

## Abstract

**Objective:**

Excessive exercise increases the production of reactive oxygen species in skeletal muscles. Sulforaphane activates nuclear factor erythroid 2‐related factor 2 (Nrf2) and induces a protective effect against oxidative stress. In a recent report, sulforaphane intake suppressed exercise‐induced oxidative stress and muscle damage in mice. However, the effect of sulforaphane intake on delayed onset muscle soreness after eccentric exercise in humans is unknown. We evaluated the effect of sulforaphane supplement intake in humans regarding the delayed onset muscle soreness (DOMS) after eccentric exercise.

**Research Methods & Procedures:**

To determine the duration of sulforaphane supplementation, continuous blood sampling was performed and *NQO1* mRNA expression levels were analyzed. Sixteen young men were randomly divided into sulforaphane and control groups. The sulforaphane group received sulforaphane supplements. Each group performed six set of five eccentric exercise with the nondominant arm in elbow flexion with 70% maximum voluntary contraction. We assessed muscle soreness in the biceps using the visual analog scale, range of motion (ROM), muscle damage markers, and oxidative stress marker (malondialdehyde; MDA).

**Results:**

Sulforaphane supplement intake for 2 weeks increased *NQO1* mRNA expression in peripheral blood mononuclear cells (PBMCs). Muscle soreness on palpation and ROM were significantly lower 2 days after exercise in the sulforaphane group compared with the control group. Serum MDA showed significantly lower levels 2 days after exercise in the sulforaphane group compared with the control group.

**Conclusion:**

Our findings suggest that sulforaphane intake from 2 weeks before to 4 days after the exercise increased *NQO1*, a target gene of Nrf2, and suppressed DOMS after 2 days of eccentric exercise.

## Graphical abstract

Sulforaphane supplement intake for 2 weeks enhances antioxidant capacity, and suppression exercise induced oxidative stress. Muscle soreness on palpation and ROM were significantly lower 2 days after exercise in the sulforaphane group compared with the control group. This result should contribute to the improvement of performance in athletes and may also increase the exercise orientation of generally healthy people.

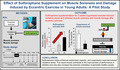

## 1 BACKGROUND

Individuals who do not exercise daily are prone to muscle pain after acute exercise, and this is one of the factors that hinders the formation of exercise habits. Myalgia that occurs a certain period after exercise is called delayed onset muscle soreness (DOMS). It can be observed as pain after exercise or on palpation of the skeletal muscle. There are several possible causes of DOMS, including increased lactate concentration (Cheung et al., 2003), muscle spasm, connective tissue and muscle damage, and inflammatory response (Armstrong, 1984), but increased skeletal muscle oxidative stress is an important factor (Maughan et al., 1989; Paschalis, Nikolaidis, Fatouros, et al., 2007; Ristow et al., 2009).

Excessive exercise increases the production of reactive oxygen species (ROS) and increases skeletal muscle oxidative stress. Oxidative stress causes damage to muscle tissue, decreased muscle contractility, and decreased exercise tolerance (Reid, 2016); thus, the suppression of ROS production is important. During exercise, the production of active oxygen increases and oxidative stress is induced. Many studies have shown that lipid peroxidation products (malonaldehyde [MDA]) are elevated in skeletal muscle and serum after transient exercise (Oh‐ishi et al., 1997; Paschalis, Nikolaidis, Giakas, et al., 2007), and there have been several studies of supplements targeting the suppression of oxidative stress (Mason et al., 2020).

The transcription factor Nrf2 (nuclear factor erythroid 2‐related factor 2) is an important master gene that responds to oxidative stress (Uruno & Motohashi, 2011). Nrf2 regulates the genes of various enzymes involved in biological defense such as heme oxygenase 1 (*HO‐1*) and NAD(P)H quinone dehydrogenase 1 (*NQO1*). On the other hand, sulforaphane (SFN), a phytochemical that activates Nrf2, induces a cytoprotective effect on oxidative stress (Guerrero‐Beltran et al., 2012; Thimmulappa et al., 2002).

In in vitro experiments, Nrf2 translocated into the nucleus due to the development of ROS in the skeletal myoblast cell line (C2C12) (Horie et al., 2015; Merry & Ristow, 2016). Oh et al. applied acute running exercise to SFN‐administered mice and investigated the effects on oxidative stress, muscle damage, and exercise tolerance in skeletal muscle after the acute running exercise (Oh et al., 2017). It was clarified that Nrf2 was activated in mouse skeletal muscle by SFN administration, and the expression level of Nrf2 target genes such as *HO‐1* and *NQO1* was increased. In addition, SFN administration suppressed oxidative stress and muscle damage and improved exercise tolerance. Recently, Bose et al. reported that SFN increased Nrf2 activity in skeletal muscle and inhibited the decline in muscle function caused by aging in mice (Bose et al., 2020). These results suggest that SFN supplementation in humans may activate Nrf2 in skeletal muscle and induce a myoprotective effect. Moreover, Lee et al. suggested that oxidative stress is one of the factors that induce DOMS (Lee et al., 2002), suggesting that activation of Nrf2 by SFN might suppress DOMS via antioxidative effect. However, the effect of SFN intake on DOMS in humans after exercise is unknown.

This pilot study aimed to evaluate the effect of SFN intake in humans with respect to the suppression of DOMS after eccentric exercise using subjective indicators and biochemical indicators.

## 2 MATERIALS AND METHODS

The ethics committee approved the study protocol, which conformed to the ethical principles of the 7th revision (2013) of the Declaration of Helsinki. The participants were asked to refrain from taking of broccoli sprouts, which are rich in SFN during the experiment. In addition, participants were instructed to refrain from (1) massaging the upper arm during exercise experiments, (2) strenuous exercise, (3) alcohol, and (4) supplements. Participants provided written informed consent and authorization for the disclosure of protected health information before enrolling in the study.

### 2.1 Study subjects (Experiment 1)

The first experiment included six healthy men without exercise habits, smoking, or medication and aimed to determine the duration of supplement intake. SFN supplements were purchased from Kagome Co. Ltd. The ingredients of the supplement are indicated in Table 1. The tablet used in this study contains 30 mg/3 tablet of SGS, a precursor of SFN, which is converted to SFN in the intestinal lumen, by myrosinase of the intestinal microflora (Fahey et al., 2001). Subjects took one tablet of SFN supplement per meal, three times a day. Blood sampling was conducted before supplement intake and weekly after supplement intake. Gene expression levels of the Nrf2 target genes (*NQO1* and *HO‐1*) in peripheral blood mononuclear cells (PBMCs) before and after supplement intake were compared. The period of the supplement intake was determined when the mRNA expression level in the PBMCs increased compared to before the intake of supplement.

**TABLE 1:** Content of sulforaphane supplement

Nutrients	Per three tablets
Calories	3 kcal
Protein	0–0.2 g
Fat	0–0.02 g
Carbohydrate	0.6 g
SGS	30 mg

Note

The SFN group took one tablet per meal (three tablets per day) during the experiment including the 2‐week pre‐exercise period, just before the exercise, just after the exercise, and post‐exercise for 4 days. SGS: glucosinolates precursor of SFN (sulforaphane glucosinolate).

### 2.2 Study subjects (Experiment 2)

The second experiment included 16 healthy men without exercise habits, smoking, or medication were included in the experiment. Sixteen young men were randomly divided into an SFN supplement group (SFN group) or a control group (CON group). There were no differences in physical characteristics between the two groups (Table 2). All subjects were instructed to avoid strenuous exercise, drinking, massage, and medication during the study duration. Before the experiment, the height, body weight, body mass index, body fat percentage, and body skeletal muscle weight were measured with a body composition analyzer (Tanita). Moreover, the maximal voluntary contraction (MVC) of the biceps was measured using Biodex system 4 (Sakai Med. Co.,). The participants started taking supplements after a 2‐week washout period after the MVC was measured. On the exercise day, subjects took supplements after arriving at the lab. Afterward, blood samples and measurements at Ex‐Pre were taken, which took 10–20 min. After that, exercise was performed within 30 min. Over the next 4 days, all body functions were analyzed within 30 min of taking the supplement. All measurements were performed by the same evaluator who was unaware of the group allocation.

**TABLE 2:** Characteristics of the subjects in the first and second experiments

First experiment							
Groups	*n*	Age	Height	Weight	BMI	%Fat	Muscle
		year	cm	kg	kg/m^2^	%	kg
	6	23.6 ± 0.4	171.9 ± 1.8	69.1 ± 3.9	23.6 ± 1.5	20.8 ± 3.17	51.3 ± 1.6

Second experiment								
Groups	*n*	Age	Height	Weight	BMI	%Fat	Muscle	MVC
		year	cm	kg	kg/m^2^	%	kg	kg
Control	8	22 ± 1	170.9 ± 1.7	61.3 ± 3.6	20.9 ± 1.2	16.8 ± 1.9	48.0 ± 1.8	16.4 ± 1.4
Sulforaphane	8	20 ± 0.8	171.7 ± 1.9	63.2 ± 2.6	21.5 ± 0.9	16.6 ± 1.5	49.8 ± 1.7	17.0 ± 1.9

Note

Values are presented as mean ± SE.

%fat, whole body fat/body weight; muscle, whole body muscle; MVC, maximal voluntary contraction.

### 2.3 Isolation of peripheral blood mononuclear cells from the blood sample

PBMCs were isolated from whole blood using lymphocyte separation medium (LSM) density gradients (MP Biomedical). Blood was placed on LSM solution and centrifuged at 200 G for 20 min to extract the lymphocyte layer (PBMCs). The PBMCs were washed with saline and centrifuged at 200 G for 10 min. The precipitate was used as the PBMC sample.

### 2.4 Real‐time quantitative polymerase chain reaction

Total RNA was extracted by adding chloroform to PBMCs containing Sepasol‐RNA I super G, with immediate mixing and centrifugation. We added 2‐propanol and centrifugation was repeated. After centrifugation, the supernatant was washed with ethanol and dried. cDNA synthesis was performed using Takara PrimeScript™ RT Master Mix (Takara Bio) using the standard protocol. Real‐time quantitative polymerase chain reaction (qPCR) was performed with cDNA and fast SYBR Green Mix (Thermo Fisher Scientific). The qPCR analysis used the CFX384™Real‐Time System (BIORAD). The following primers were used: *NQO1*—Forward (F), 5'‐CTGATCGTACTGGCTCACTC‐3', Reverse (R), 5'‐AACAGACTCGGCAGGATAC‐3', *HO‐1*—Forward, 5'‐CCAGGCAGAGAATGCTGAGT‐3', Reverse, 5'‐GTAGACAGGGGCGAAGACTG‐3', and *GAPDH*—Forward, 5'‐AGGTGAAGGTCGGAGTCA‐3', Reverse, 5'‐GGTCATTGATGGCAACAA‐3'. The mRNA expression levels were normalized to that of the gene encoding GAPDH.

### 2.5 Exercise protocol

**FIGURE 1:**
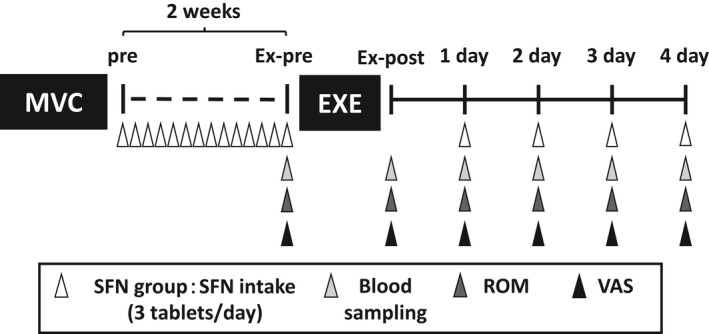
A schematic illustrating the experimental protocol and time course of the study. The arrowheads indicate the timing of SFN intake or analysis during the experiment. Ex‐post: just after exercise; Ex‐pre: before exercise; Pre: before sulforaphane intake; 1 day: 1 day after exercise; 2 day: 2 days after exercise; 3 day: 3 days after exercise; 4 day: 4 days after exercise. EXE, eccentric exercise; MVC, maximal voluntary contraction; ROM, range of motion; SFN, sulforaphane; VAS, visual analog scale

The exercise experiment comprised before exercise, immediately after exercise, and 1, 2, 3, and 4 days after the exercise (Figure 1). The exercise, blood sampling, and measurement for all participants were on an empty stomach early in the morning (6:00–8:30 a.m.). During the measurements, subjects gathered in the laboratory and only subjects of the SFN group took the SFN supplement. First, subjects underwent blood sampling and were then assessed for muscle soreness using the visual analog scale (VAS) and range of motion (ROM) of the elbow joint before exercise.

The exercise protocol was an eccentric exercise of the brachial flexor muscle group of the nondominant arm (Ra et al., 2013). Exercise intensity was set to 70% MVC and was performed for 5 s × 5 repetitions × 6 sets. There was a 2‐min break between sets. The exercise was performed according to an electronic metronome (60 beats/min), and we provided assistance when the subjects could not maintain the rhythm by themselves. After an eccentric contraction of 5 s, we took 3 s to achieve the correct arm position; thereafter, we started at the same position again with the subject relaxed.

### 2.6 Evaluation of muscle soreness and muscle damage

Subjective muscle soreness in the biceps muscle was evaluated by VAS, which consisted of a 100‐mm straight line with "no pain" at the left end and "extreme pain" at the right end. Muscle soreness was assessed before exercise, immediately after exercise, and from Day 1 to Day 4 based on the subject's subjective judgment. Two types of evaluation for muscle soreness were performed: palpation and spontaneous maximal extension (Ra et al., 2013). Muscle soreness on palpation was evaluated when the evaluator palpated the muscle belly of the biceps for 3 s. Muscle soreness on spontaneous maximal extension was evaluated when the subjects extended the elbow joint. VAS was calculated as score of each days minus Ex‐pre. In order to match the assessments, the same evaluator was used for all subjects.

### 2.7 Evaluation of range of motion

ROM in the elbow joint, which reflects muscle soreness and damage, was measured using a goniometer (TAKASE MED). Starting from the lateral superior condyle of the humerus, we aligned both axes of the goniometer with the acromion and radial styloid process, and set the maximum extension and maximum flexion within a pain‐free range. ROM was calculated as maximum extension angle minus maximum flexion angle. Maximum flexion elbow joint angle and maximum extension elbow joint angle were calculated as each days of angle minus Ex‐pre of angle. The ROM was measured twice and the average value was calculated.

### 2.8 Blood parameters

Blood samples were collected from the antecubital vein. After the blood sampling, samples were requested from the Tsukuba i‐Laboratory LLP to measure muscle damage makers (CK; creatine kinase, LDH; lactate dehydrogenase, ALD; aldolase, and AST; aspartate aminotransferase). After measuring, the serums were stored at −80℃ until they were needed for measurement.

### 2.9 Measurement of oxidative stress marker

Lipid peroxidation (Malondialdehyde; MDA) is an indicator of exercise‐induced oxidative stress. To evaluate oxidative stress after eccentric exercise, we measured MDA in the serum. Serum MDA was measured using the standard protocol of the TBARS assay kit (Cayman Chemical). Briefly, the MDA–thiobarbituric acid (TBA) adduct formed by the reaction of MDA and TBA under high temperature (90–100°C) and acidic conditions were measured with the Varioskan microplate reader (Thermo Fisher Scientific). An absorbance of 540 nm was used.

### 2.10 Statistical analysis

Statistical analyses were performed with SPSS version 25 for Windows (IBM). All data are expressed as mean ± standard error (SE). Comparisons between mRNA levels were performed using one‐way analysis of variance followed by Tukey's multiple comparison. The Mann–Whitney *U* test was used to compare the two groups. The Wilcoxon signed‐rank test was used to compare the Ex‐Pre or Ex‐Post. A *p* value less than 0.05 was considered statistically significant.

## 3 RESULTS

**FIGURE 2:**
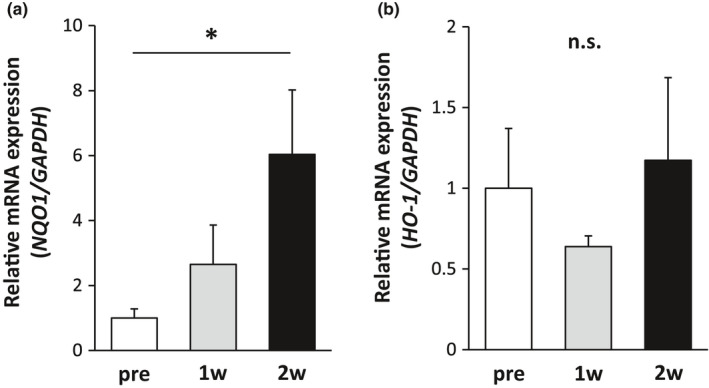
Changes in Nrf2 target genes due to sulforaphane supplementation. mRNA expression of (a) *NQO1 and* (b) *HO‐1* in PBMCs. Asterisk indicates significant differences between groups (*p* < 0.05, one‐way ANOVA followed by Tukey's multiple comparison) (*n* = 8)

The mRNA expression levels of *NQO1* and *HO‐1*, the target genes of Nrf2, in PBMCs before 1 week, and 2 weeks after SFN intake are shown in Figure 2. *NQO1* was significantly higher at 2 weeks than before SFN intake, while *HO‐1* was not significantly different between these two periods. Based on these results, we decided on a 2‐week SFN intake period and conducted the eccentric exercise experiment.

**FIGURE 3:**
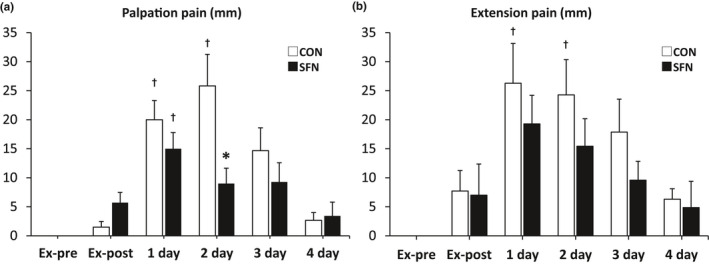
Normalized changes in the visual analog scale (VAS). (a) Change in muscle soreness at palpation. (b) Change in muscle soreness on extension. Both changes were analyzed by VAS. Calculated by score of each days––Ex‐pre. Asterisk indicates significant differences between the groups (*p* < 0.05, Mann–Whitney *U* test) (*n* = 8). Dagger indicates significant differences with Ex‐post (*p* < 0.05, Wilcoxon signed‐rank test). CON, control group; Ex‐pre, before exercise; SFN, sulforaphane group; 1 day: 1 day after exercise; 2 day: 2 days after exercise; 3 day: 3 days after exercise; 4 day: 4 days after exercise

DOMS was induced by applying a load of 70% MVC and performing an extensional exercise. Subjective muscle soreness was analyzed by VAS. The VAS on palpation of CON groups peaked at 2 days after exercise. On the other hand, the VAS of SFN group peaked at 1 day after exercise. The values in CON group were significantly higher at 1 and 2 days than immediately after exercise (Ex‐post). The SFN group showed a significantly lower VAS on palpation at 2 days after exercise than the CON group (Figure 3a). The VAS on extension pain peaked at 1 day after exercise in the CON and SFN groups, but the pain was observed at 1 and 2 days after exercise only in the CON group (Figure 3b). There was no significant difference between the CON and the SFN groups.

**FIGURE 4:**
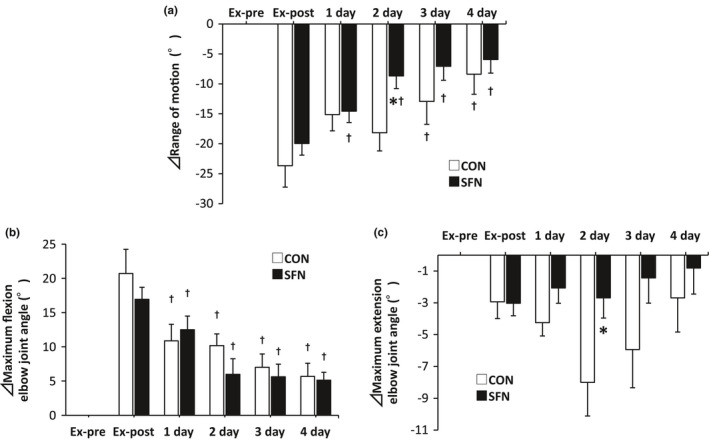
Changes in range of motion. (a) Range of motion. (b) Elbow joint flexion angle. (c) Elbow joint extension angle. Asterisk indicates significant differences between the groups (*p* < 0.05, Mann–Whitney *U* test) (*n* = 8). Dagger indicates significant differences with Ex‐post (*p* < 0.05, Wilcoxon signed‐rank test). CON: control group; Ex‐post: just after exercise; Ex‐pre: before exercise; SFN: sulforaphane group; 1 day: 1 day after exercise; 2 day: 2 days after exercise; 3 day: 3 days after exercise; 4 day: 4 days after exercise

The ROM of the elbow joint, the angle at elbow flexion, and the angle at elbow extension from before to 4 days after the exercise experiment are shown in Figure 4a–c. The ROM of the elbow joint within the pain‐free range was significantly smaller in the SFN group than in the CON group at 2 days after exercise (Figure 4a). Compared to the Ex‐post, the ROM of the CON group showed a significant difference after 3 days of exercise, while that of the SFN group showed a significant difference from 1 day after exercise. In the analysis of the elbow flexion and extension angles, the peak flexion angle appeared immediately after the exercise and then gradually decreased, but there was no significant difference between the groups (Figure 4b). However, the decrease in the extension angle peaked at 2 days after exercise, but this peak was suppressed by SFN intake (Figure 4c).

**FIGURE 5:**
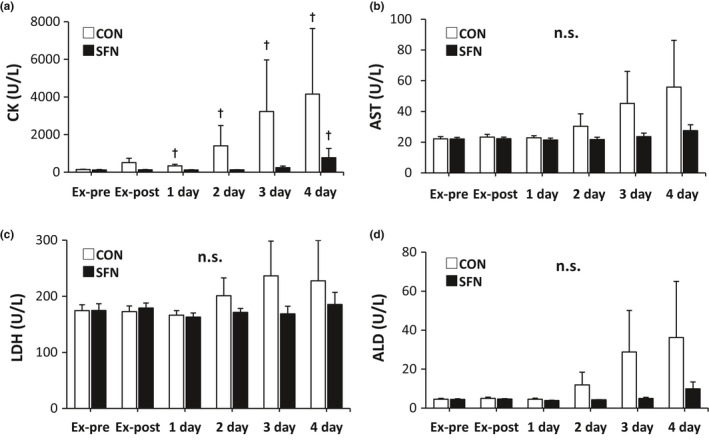
Changes in serum muscle damage markers. (a) CK activity, (b) AST activity, (c) LDH activity, and (d) ALD activity. (*n* = 8). Dagger indicates significant differences with Ex‐pre (*p* < 0.05, Wilcoxon signed‐rank test). ALD, aldolase; AST, aspartate aminotransferase; CK, creatine kinase; CON, control group; Ex‐post, just after exercise; Ex‐pre, before exercise; LDH, lactate dehydrogenase; SFN, sulforaphane group; 1 day: 1 day after exercise; 2 day: 2 days after exercise; 3 day: 3 days after exercise; 4 day: 4 days after exercise

The changes in muscle damage markers (CK, AST, LDH, and ALD) from before exercise to 4 days after exercise are shown in Figure 5a–d. Compared to the Ex‐pre, the CK activity in the serum of the CON group showed a significant increase after 1 day of exercise, while that of the SFN group showed a significant increase at 4 days after exercise. The CK activity in the serum of the SFN group tended to be lower than that of the CON group after 2 days of exercise, but there was no significant difference between the CON and SFN groups (*p* = 0.081, Figure 5a). The peak values of AST, LDH, and ALD occurred 3–4 days after exercise, but there was no significant difference between the CON and SFN groups.

**FIGURE 6:**
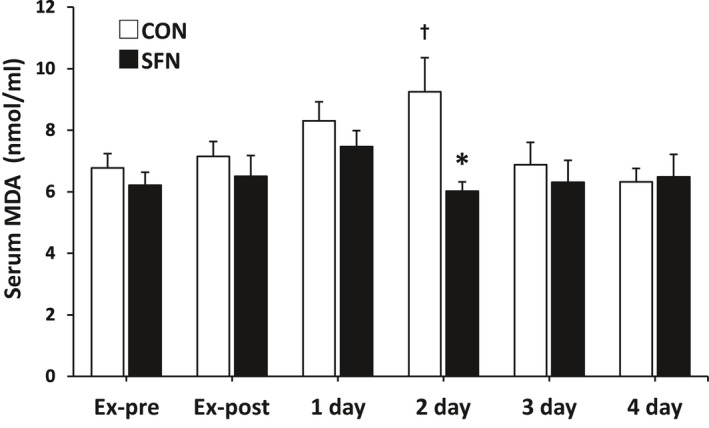
Changes in serum oxidative stress marker. Malondialdehyde concentration in serum. Asterisk indicates significant differences between the groups (*p* < 0.05, Mann–Whitney *U* test) (*n* = 8). Dagger indicates significant differences with Ex‐pre (*p* < 0.05, Wilcoxon signed‐rank test). CON: control group; Ex‐post: just after the exercise; Ex‐pre: before the exercise; SFN: sulforaphane group; 1 day: 1 day after exercise; 2 day: 2 days after exercise; 3 day: 3 days after exercise; 4 day: 4 days after exercise; MDA, malondialdehyde

The changes in oxidative stress markers (MDA) are shown in Figure 6. Compared to the Ex‐pre, the serum MDA concentration showed significant increase after 2 days of exercise only in the CON group. In the SFN group, the serum MDA concentration was significantly lower at 2 days after exercise compared to the CON group.

## 4 DISCUSSION

In this study, the continuation of SFN intake from 2 weeks before and up to 4 days after the eccentric exercise suppressed exercise‐induced oxidative stress and inhibited muscle soreness and muscle damage after eccentric exercise. To our knowledge, the present study is the first to analyze the effects of SFN in humans; SFN was shown to be effective in mice for muscle damage (Oh et al., 2017).

In this study, 2 weeks of SFN intake resulted in a significant increase in the mRNA expression of *NQO1*, a target gene of Nrf2, but no change in the mRNA expression of *HO‐1* was noted. *NQO1* has been used to assess Nrf2 activity (Malaguti et al., 2009; Oh et al., 2017; Ushida et al., 2015). Ushida et al. measured serum NQO1 activity before and 24 h after the ingestion of 30 and 60 mg of glucoraphanin (the precursor of SFN; SGS) (Ushida et al., 2015). Serum NQO1 activity was significantly higher in the 30 and 60 mg groups at 24 h after intake than before intake. Like the above study, ours reported a significant duration‐dependent increase in serum *NQO1* mRNA expression. Recently, Liu et al. reported that when SFN was added to the PBMCs of healthy subjects in ex vivo experiments, *NQO1* expression was increased, while *HO‐1* was not increased at a low SFN concentration (0.5 µM) (Liu et al., 2020). However, when 2 or 5 µM of SFN was added to the PBMCs, both *NQO1* and *HO‐1* gene expression were increased. The concentration of the SFN supplement may be a reason why the amount of supplementation used in our protocol (30 mg/ 1 day, for 2 weeks) did not increase *HO‐1* expression. Based on our first experiment, we speculated that SFN intake for 2 weeks was sufficient to activate Nrf2, because the mRNA expression of *NQO1* was increased.

We found that muscle soreness on palpation was suppressed 2 days after exercise in the SFN group compared with the CON group. Lau et al. performed 6 × 10 sets of maximal eccentric contractions in an upper arm flexion group of healthy men without exercise habits and observed a change in pain on palpation from pre‐exercise to 5 days post‐exercise (Lau et al., 2015). The pain on palpation reached its peak 1–2 days after exercise and recovered to baseline 5 days after exercise. The CON group in this study showed similar changes to those in this previous study, indicating that the induction of DOMS by exercise load could be reproduced. However, the pain on palpation in the SFN group peaked after 1 day of exercise and was lower than that in the CON group after 2 days of exercise. The peak in the SFN group may have been an apparent peak on Day 1 as a result of pain suppression after 2 days. The difference between the groups after 2 days of exercise in this study may be due to the suppression of endogenous metabolic responses (e.g., oxidative stress and inflammation) via Nrf2, which suppressed the peak after 2 days (Kobayashi et al., 2016).

The decrease in elbow ROM at 2 days after exercise was suppressed in the SFN group compared with the CON group. The decrease in the elbow extension angle was suppressed in the SFN group, and there was no significant difference in the elbow flexion angle between the groups. The decrease in elbow ROM may be due to the suppression of the joint angle in extension rather than in flexion. ROM was decreased by DOMS (Ra et al., 2018). In the present study, ROM was assessed by range of motion of the elbow joint within the pain‐free range, and it is thought that the suppression of DOMS was involved in the maintenance of ROM.

We measured serum CK, LDH, AST, and ALD activities because they are markers of muscle damage. Furthermore, DOMS is associated with muscle damage, and when muscle damage occurs, CK, LDH, ALD, and AST, which are enzymes present in muscle cells, are released into the blood (Bassit et al., 2010; Clarkson et al., 1992; Cooke et al., 2009; Nosaka et al., 2002a, 2002b, 2005). In particular, CK activity has been used as a marker of muscle damage in many studies, and the peak increase in CK activity after exercise occurs 2–4 days after exercise (Manfredi et al., 1991). In an upper arm flexion group, CK activity in the blood peaked at 4 days after exercise (Nosaka et al., 2002a; Tanabe et al., 2015), and in our SFN group, CK activity peaked at 4 days after exercise. In the SFN group, there was a trend toward a lower value at 2 days after exercise (*p* = 0.081) compared with the CON group, suggesting that muscle damage might have been suppressed.

During exercise, the production of ROS markedly increases and oxidative stress is induced. Many studies have shown that TBARS increases in the skeletal muscle, myocardium, liver, and plasma after transient exercise (Paschalis, Nikolaidis, Giakas, et al., 2007). In addition, TBARS, an oxidative stress marker after acute exercise, increases significantly 2 days after exercise (Paschalis, Nikolaidis, Fatouros, et al., 2007; Paschalis, Nikolaidis, Giakas, et al., 2007; Ra et al., 2013). In the present study, TBARS showed a peak value after 2 days of exercise, and the value was significantly lower in the SFN group. In the present study, TBARS levels peaked after 2 days of exercise and were significantly lower in the SFN group, suggesting that SFN intake suppressed exercise‐induced oxidative stress. SFN intake may protect the balance of antioxidant capacity and suppress the excessive oxidative stress caused by exercise.

These results showed that the continuation of SFN intake from 2 weeks before and up to 4 days after the eccentric exercise suppressed muscle soreness and muscle damage after elongated exercise. We suggest that the suppression of exercise‐induced oxidative stress by SFN was involved in this process. This study was limited by the lack of a placebo control and the sample size. Additionally, we indirectly assessed Nrf2 activation using *NQO1* mRNA expression in PBMCs. In the future, it is desirable to directly analyze the activation of Nrf2 in skeletal muscle after supplementation. Further studies are needed to clarify our findings.

## CONFLICT OF INTEREST

The authors declare that they have no competing interest.

## AUTHORS' CONTRIBUTIONS

Conceptualization: S. Komine and S. Oh. Methodology: S. Komine, I. Miura, and N. Miyashita. Formal analysis and investigation: S. Komine, I. Miura, N. Miyashita, and J. Shoda. Writing‐original draft preparation: S. Komine and I. Miura. Writing‐review and editing: S. Komine and I Miura. Funding acquisition: S. Komine. Resources: S. Komine. Supervision: H. Ohmori.

## References

[phy215130-bib-0001] Armstrong, R. B. (1984). Mechanisms of exercise‐induced delayed onset muscular soreness: A brief review. Medicine and Science in Sports and Exercise, 16, 529–538. 6392811

[phy215130-bib-0002] Bassit, R. A. , Pinheiro, C. H. , Vitzel, K. F. , Sproesser, A. J. , Silveira, L. R. , & Curi, R. (2010). Effect of short‐term creatine supplementation on markers of skeletal muscle damage after strenuous contractile activity. European Journal of Applied Physiology, 108, 945–955. 19956970 10.1007/s00421-009-1305-1

[phy215130-bib-0003] Bose, C. , Alves, I. , Singh, P. , Palade, P. T. , Carvalho, E. , Borsheim, E. , Jun, S. R. , Cheema, A. , Boerma, M. , Awasthi, S. , & Singh, S. P. (2020). Sulforaphane prevents age‐associated cardiac and muscular dysfunction through Nrf2 signaling. Aging Cell, 19, e13261. 33067900 10.1111/acel.13261 PMC7681049

[phy215130-bib-0004] Cheung, K. , Hume, P. , & Maxwell, L. (2003). Delayed onset muscle soreness : Treatment strategies and performance factors. Sports Medicine (Auckland, N. Z.), 33, 145–164. 10.2165/00007256-200333020-00005 12617692

[phy215130-bib-0005] Clarkson, P. M. , Nosaka, K. , & Braun, B. (1992). Muscle function after exercise‐induced muscle damage and rapid adaptation. Medicine and Science in Sports and Exercise, 24, 512–520. 1569847

[phy215130-bib-0006] Cooke, M. B. , Rybalka, E. , Williams, A. D. , Cribb, P. J. , & Hayes, A. (2009). Creatine supplementation enhances muscle force recovery after eccentrically‐induced muscle damage in healthy individuals. Journal of the International Society of Sports Nutrition, 6, 13. 19490606 10.1186/1550-2783-6-13 PMC2697134

[phy215130-bib-0007] Fahey, J. W. , Zalcmann, A. T. , & Talalay, P. (2001). The chemical diversity and distribution of glucosinolates and isothiocyanates among plants. Phytochemistry, 56, 5–51. 11198818 10.1016/s0031-9422(00)00316-2

[phy215130-bib-0008] Guerrero‐Beltran, C. E. , Calderon‐Oliver, M. , Pedraza‐Chaverri, J. , & Chirino, Y. I. (2012). Protective effect of sulforaphane against oxidative stress: Recent advances. Experimental and Toxicologic Pathology, 64, 503–508. 21129940 10.1016/j.etp.2010.11.005

[phy215130-bib-0009] Horie, M. , Warabi, E. , Komine, S. , Oh, S. , & Shoda, J. (2015). Cytoprotective role of Nrf2 in electrical pulse stimulated C2C12 Myotube. PLoS One, 10, e0144835. 26658309 10.1371/journal.pone.0144835 PMC4681703

[phy215130-bib-0010] Kobayashi, E. H. , Suzuki, T. , Funayama, R. , Nagashima, T. , Hayashi, M. , Sekine, H. , Tanaka, N. , Moriguchi, T. , Motohashi, H. , Nakayama, K. , & Yamamoto, M. (2016). Nrf2 suppresses macrophage inflammatory response by blocking proinflammatory cytokine transcription. Nature Communications, 7, 11624. 10.1038/ncomms11624 PMC4879264 27211851

[phy215130-bib-0011] Lau, W. Y. , Blazevich, A. J. , Newton, M. J. , Wu, S. S. , & Nosaka, K. (2015). Assessment of muscle pain induced by elbow‐flexor eccentric exercise. Journal of Athletic Training, 50, 1140–1148. 26523661 10.4085/1062-6050-50.11.05 PMC4732393

[phy215130-bib-0012] Lee, J. , Goldfarb, A. H. , Rescino, M. H. , Hegde, S. , Patrick, S. , & Apperson, K. (2002). Eccentric exercise effect on blood oxidative‐stress markers and delayed onset of muscle soreness. Medicine and Science in Sports and Exercise, 34, 443–448. 11880808 10.1097/00005768-200203000-00010

[phy215130-bib-0013] Liu, H. , Zimmerman, A. W. , Singh, K. , Connors, S. L. , Diggins, E. , Stephenson, K. K. , Dinkova‐Kostova, A. T. , & Fahey, J. W. (2020). Biomarker exploration in human peripheral blood mononuclear cells for monitoring sulforaphane treatment responses in autism spectrum disorder. Scientific Reports, 10, 5822. 32242086 10.1038/s41598-020-62714-4 PMC7118069

[phy215130-bib-0014] Malaguti, M. , Angeloni, C. , Garatachea, N. , Baldini, M. , Leoncini, E. , Collado, P. S. , Teti, G. , Falconi, M. , Gonzalez‐Gallego, J. , & Hrelia, S. (2009). Sulforaphane treatment protects skeletal muscle against damage induced by exhaustive exercise in rats. Journal of Applied Physiology, 1985(107), 1028–1036. 10.1152/japplphysiol.00293.2009 19713431

[phy215130-bib-0015] Manfredi, T. G. , Fielding, R. A. , O'Reilly, K. P. , Meredith, C. N. , Lee, H. Y. , & Evans, W. J. (1991). Plasma creatine kinase activity and exercise‐induced muscle damage in older men. Medicine and Science in Sports and Exercise, 23, 1028–1034. 1943622

[phy215130-bib-0016] Mason, S. A. , Trewin, A. J. , Parker, L. , & Wadley, G. D. (2020). Antioxidant supplements and endurance exercise: Current evidence and mechanistic insights. Redox Biology, 35, 101471. 32127289 10.1016/j.redox.2020.101471 PMC7284926

[phy215130-bib-0017] Maughan, R. J. , Donnelly, A. E. , Gleeson, M. , Whiting, P. H. , Walker, K. A. , & Clough, P. J. (1989). Delayed‐onset muscle damage and lipid peroxidation in man after a downhill run. Muscle and Nerve, 12, 332–336. 2770784 10.1002/mus.880120412

[phy215130-bib-0018] Merry, T. L. , & Ristow, M. (2016). Nuclear factor erythroid‐derived 2‐like 2 (NFE2L2, Nrf2) mediates exercise‐induced mitochondrial biogenesis and the anti‐oxidant response in mice. Journal of Physiology, 594, 5195–5207. 10.1113/JP271957 PMC5023720 27094017

[phy215130-bib-0019] Nosaka, K. , Newton, M. , & Sacco, P. (2002a). Delayed‐onset muscle soreness does not reflect the magnitude of eccentric exercise‐induced muscle damage. Scandinavian Journal of Medicine and Science in Sports, 12, 337–346. 12453160 10.1034/j.1600-0838.2002.10178.x

[phy215130-bib-0020] Nosaka, K. , Newton, M. , & Sacco, P. (2002b). Responses of human elbow flexor muscles to electrically stimulated forced lengthening exercise. Acta Physiologica Scandinavica, 174, 137–145. 11860376 10.1046/j.1365-201X.2002.00936.x

[phy215130-bib-0021] Nosaka, K. , Newton, M. , Sacco, P. , Chapman, D. , & Lavender, A. (2005). Partial protection against muscle damage by eccentric actions at short muscle lengths. Medicine and Science in Sports and Exercise, 37, 746–753. 15870627 10.1249/01.mss.0000162691.66162.00

[phy215130-bib-0022] Oh, S. , Komine, S. , Warabi, E. , Akiyama, K. , Ishii, A. , Ishige, K. , Mizokami, Y. , Kuga, K. , Horie, M. , Miwa, Y. , Iwawaki, T. , Yamamoto, M. , & Shoda, J. (2017). Nuclear factor (erythroid derived 2)‐like 2 activation increases exercise endurance capacity via redox modulation in skeletal muscles. Scientific Reports, 7, 12902. 29018242 10.1038/s41598-017-12926-y PMC5635018

[phy215130-bib-0023] Oh‐Ishi, S. , Kizaki, T. , Ookawara, T. , Sakurai, T. , Izawa, T. , Nagata, N. , & Ohno, H. (1997). Endurance training improves the resistance of rat diaphragm to exercise‐induced oxidative stress. American Journal of Respiratory and Critical Care Medicine, 156, 1579–1585. 9372679 10.1164/ajrccm.156.5.96-11035

[phy215130-bib-0024] Paschalis, V. , Nikolaidis, M. G. , Fatouros, I. G. , Giakas, G. , Koutedakis, Y. , Karatzaferi, C. , Kouretas, D. , & Jamurtas, A. Z. (2007). Uniform and prolonged changes in blood oxidative stress after muscle‐damaging exercise. In Vivo, 21, 877–883. 18019428

[phy215130-bib-0025] Paschalis, V. , Nikolaidis, M. G. , Giakas, G. , Jamurtas, A. Z. , Pappas, A. , & Koutedakis, Y. (2007). The effect of eccentric exercise on position sense and joint reaction angle of the lower limbs. Muscle and Nerve, 35, 496–503. 17221879 10.1002/mus.20723

[phy215130-bib-0026] Ra, S. G. , Miyazaki, T. , Ishikura, K. , Nagayama, H. , Komine, S. , Nakata, Y. , Maeda, S. , Matsuzaki, Y. , & Ohmori, H. (2013). Combined effect of branched‐chain amino acids and taurine supplementation on delayed onset muscle soreness and muscle damage in high‐intensity eccentric exercise. Journal of the International Society of Sports Nutrition, 10, 51. 24195702 10.1186/1550-2783-10-51 PMC3827986

[phy215130-bib-0027] Ra, S. G. , Miyazaki, T. , Kojima, R. , Komine, S. , Ishikura, K. , Kawanaka, K. , Honda, A. , Matsuzaki, Y. , & Ohmori, H. (2018). Effect of BCAA supplement timing on exercise‐induced muscle soreness and damage: A pilot placebo‐controlled double‐blind study. Journal of Sports Medicine and Physical Fitness, 58, 1582–1591. 10.23736/S0022-4707.17.07638-1 28944645

[phy215130-bib-0028] Reid, M. B. (2016). Reactive oxygen species as agents of fatigue. Medicine and Science in Sports and Exercise, 48, 2239–2246. 27285492 10.1249/MSS.0000000000001006

[phy215130-bib-0029] Ristow, M. , Zarse, K. , Oberbach, A. , Kloting, N. , Birringer, M. , Kiehntopf, M. , Stumvoll, M. , Kahn, C. R. , & Bluher, M. (2009). Antioxidants prevent health‐promoting effects of physical exercise in humans. Proceedings of the National Academy of Sciences of the United States of America, 106, 8665–8670. 19433800 10.1073/pnas.0903485106 PMC2680430

[phy215130-bib-0030] Tanabe, Y. , Maeda, S. , Akazawa, N. , Zempo‐Miyaki, A. , Choi, Y. , Ra, S. G. , Imaizumi, A. , Otsuka, Y. , & Nosaka, K. (2015). Attenuation of indirect markers of eccentric exercise‐induced muscle damage by curcumin. European Journal of Applied Physiology, 115, 1949–1957. 25921600 10.1007/s00421-015-3170-4 PMC4536282

[phy215130-bib-0031] Thimmulappa, R. K. , Mai, K. H. , Srisuma, S. , Kensler, T. W. , Yamamoto, M. , & Biswal, S. (2002). Identification of Nrf2‐regulated genes induced by the chemopreventive agent sulforaphane by oligonucleotide microarray. Cancer Research, 62, 5196–5203. 12234984

[phy215130-bib-0032] Uruno, A. , & Motohashi, H. (2011). The Keap1‐Nrf2 system as an in vivo sensor for electrophiles. Nitric Oxide, 25, 153–160. 21385624 10.1016/j.niox.2011.02.007

[phy215130-bib-0033] Ushida, Y. , Suganuma, H. , & Yanaka, A. (2015). Low‐dose of the sulforaphane precursor glucoraphanin as a dietary supplement induces chemoprotective enzymes in humans. Food and Nutrition Sciences, 06, 1603–1612.

